# Lentivirus vector-mediated gene transduction of CNGRC peptide in rat adipose stem cells

**DOI:** 10.3892/mmr.2014.3043

**Published:** 2014-12-03

**Authors:** DU MENG, RUI LIU, LI PEI, LEI HOU, QIAN NING, QING YU, LU FENG, XINHAN ZHAO

**Affiliations:** Department of Oncology, First Affiliated Hospital of Medical School of Xi’an Jiaotong University, Xi’an, Shaanxi 710061, P.R. China

**Keywords:** adipose stem cells, cell surface markers, green fluorescence protein, lentivirus vector, CNGRC peptide

## Abstract

The aim of the present study was to investigate the feasibility of lentiviral-mediated Cys-Asn-Gly-Arg-Cys (CNGRC) peptide gene transduction in adipose stem cells. Adipose stem cells were prepared using enzymatic digestion and repeated adherence methods and identified in culture by immunofluorescence staining of surface markers. The pluripotency of the cultured adipose stem cells was confirmed by their induced differentiation into bone and fat cells. Following polymerase chain reaction amplification, the gene sequence for the CNGRC peptide was cloned into a lentiviral vector, which was then co-transfected into 293T cells with packaging plasmids Helper 1.0 and Helper 2.0. The lentiviruses carrying the CNGRC peptide gene were then harvested and used to transfect adipose stem cells. Following transduction, expression of CNGRC in adipose stem cells was detected using western blot analysis. Adipose stem cells in culture were successfully induced to differentiate into adipocytes and osteoblasts and the lentiviral vector containing CNGRC-3Flag-EGFP was successfully constructed. Following transduction, western blot analysis and immunofluorescence staining demonstrated expression of the CNGRC protein in adipose stem cells. This suggested that adipose stem cell lines expressing the CNGRC peptide were successfully established.

## Introduction

Vascular niches of cancer stem cells formed by microvessels in a tumor have been considered a cause of local tumor recurrence and metastasis (1,2). Therefore, microvessels in stem cell niches constitute a new target for cancer therapy and destruction of certain clusters of microvessels may assist in eliminating cancer stem cells. Since stem cells can be recruited to stem cell niches, a process termed stem cell homing, the use of stem cells as vectors for gene therapy has become an important topic of discussion in previous years (3).

Adipose stem cells (ADSCs) isolated from adipose tissue have previously been identified in adults (4) and have phenotypes and differentiation potentials similar to those of bone marrow mesenchymal stem cells (5). Numerous studies have demonstrated that ADSCs possess an active homing capacity for multiple types of cancer, including ovarian cancer, glioma and metastatic lung cancer (4,6–8). Additionally, ADSCs are easily isolated, cultured and transfected *in vitro* and can survive following transplantation into animals (3). Therefore, ADSCs are good candidates for transporting anti-angiogenesis drugs in gene therapy as they can migrate to the microvessels of cancer stem cell niches, destroy the microvessels and ultimately eliminate the cancer stem cells.

In a previous study, a DNA sequence expressing Asn-Gly-Arg (NGR) and the cyclic peptide Cys-Asn-Gly-Arg-Cys (CNGRC) was designed that could specifically penetrate breast cancer cells (9). Additionally, *in vitro* experiments have indicated that the CNGRC peptide has antitumor activity (9). NGR is a tri-peptide motif that can specifically bind to vascular endothelial cells of newly generated blood vessels and such binding is mediated by amino peptidase N (CD13). CD13 is a transmembrane protein with a molecular weight of 140,000 Da. It is mainly expressed in solid tumors and the blood vessels of new tumors, but is rarely expressed in normal blood vessels. NGR peptide not only binds to new vessels through CD13, but can also undergo deamination of asparagine to form the isomer, isoDGR. isoDGR can regulate adhesion and proliferation of epithelial cells and has a higher affinity than NGR for blood vessels in new tumors (10). Following binding, NGR peptide can be internalized into vascular endothelial cells by receptor-mediated endocytosis. Based on this principle, NGR peptide can carry compounds and particles, including cytotoxic drugs, cytokines, virus particles and fluorescent compounds to new vessels, which can markedly improve the efficacy of targeted therapy (11). A number of studies have used NGR motif containing peptides for ligand-mediated imaging and treatment of tumors with new blood vessels (12–15). For example, Corti (16) *et al* used doxorubicin (DOX)-NGR conjugates to treat human metastatic tumors in nude mice. Compared with free DOX, DOX-peptide conjugates not only improved the efficacy of breast cancer treatment, but also significantly reduced toxicity to the liver and heart. Curnis *et al* (17) demonstrated that use of tumor necrosis factor-NGR conjugates improved drug permeability and the therapeutic effects of tumor necrosis factor by 8–10-fold. However, stem cells expressing NGR peptide and its conjugates were not available for assessment.

To establish an NGR expressing stem cell line, it is essential to select a gene delivery vector that can stably express CNGRC peptide for a long period of time. Eukaryotic viral vectors, including adenovirus vectors, adeno-associated vectors, retrovirus vectors and lentiviral vectors have high transduction efficiencies and evident targeting capacities and are most commonly used in gene therapy. However, retroviral vectors can only infect dividing cells and cannot accommodate DNA fragments >8 kb (18). Additionally, adenovirus vectors cannot achieve long-term expression of exogenous genes and repeated application of the adenovirus can lead to immune responses (19). By contrast, the human immunodeficiency virus derived lentiviral vector can infect dividing cells and non-dividing cells and produce long-term stable expression of exogenous genes, without inducing an immune response (20). Compared with other conventional transduction methods, including liposome transduction, calcium phosphate transduction and electroporation, use of a lentivirus can also efficiently infect cells that are difficult to transfect, including nerve cells, myocardial cells, endothelial cells and cells grown in suspension (21,22). Therefore, lentiviral vectors are the most satisfactory gene transfer vectors for use in gene therapy.

In the present study, an enzymatic digestion and repeat adherence methods were used to isolate highly purified ADSCs and the feasibility of using a lentiviral vector to express CNGRC peptide in ADSCs was investigated.

## Materials and methods

### Animal care and maintenance

Sprague Dawley (SD) rats aged 3 months and weighing 180–250 g were maintained at the Experimental Animal Center of Medical School of Xi’an Jiaotong University (Xi’an, China). All experimental manipulations were performed in accordance with general guidelines of the Association for Assessment and Accreditation of Laboratory Animal Care. The Animal Care and Use Committee of Xi’an Jiaotong University reviewed and approved all animal-related procedures.

### Isolation and culture of primary ADSCs

Bilateral samples of inguinal subcutaneous adipose tissue (400–600 ml) were collected from adult male SD rats. The samples were washed, cut into sections (1^3^ mm) and digested in 0.1% collagenase I at 37°C on a shaker (100 × g) for 60 min. Suspended fat was removed by centrifuging twice at 1,200 × g for 12 min. The cells were then filtered and suspended in Dulbecco’s modified Eagle’s medium (DMEM)/F12 containing 10% fetal bovine serum and penicillin/streptavidin and cultured for 72 h at 37°C, in a 5% CO_2_ humidified incubator (Thermo Fisher Scientific, Rockford, MA, USA). After 72 h, non-adherent red blood cells were removed by changing the culture medium. Following reaching 70–80% confluence, the fat cells were passaged at a ratio of 1:2 into two T-25 cell culture flasks. The cells were then cultured at 37°C in a 5% CO_2_ humidified culture container. The culture medium was changed every 2–3 days.

### Immunofluorescence staining

Surface markers CD29, CD34, CD106 and CD90 were used to identify ADSCs. Following reaching 80% confluence, at the third passage ADSCs were stained using fluorescence-labeled antibodies, phycoerythrin (PE)-conjugated mouse-anti-rat CD106 monoclonal antibody, PE mouse-anti-rat CD34 monoclonal antibody, PE mouse-anti-rat CD90 monoclonal antibody and PE mouse-anti-rat CD29 monoclonal antibody (1:5,000; BioLegend, San Diego, CA, USA). Cells were visualized and images were captured using an inverted fluorescence microscope (ECLIPSE Ti; Nikon, Tokyo, Japan).

### Adipogenic induction

ADSCs at the third passage were induced by addition of an adipogenic inducer solution containing 10% fetal bovine serum, 1 μmol/l dexamethasone, 0.5 mmol/l 3-isobutyl-1-methyl xanthine and 1 μmol/l indomethacin in DMEM/F12. Control cells were cultured in DMEM/F12 containing 10% fetal bovine serum. Cells were maintained at 37°C in a 5% CO_2_ incubator and the medium was changed every 3 days. Following a 2 week period of induction, the cells were stained with Oil Red O and observed under an inverted microscope (TMS; Nikon).

### Osteogenic induction

ADSCs at the third passage were induced by addition of induction media containing 1 μmol/l dexamethasone, 0.5 mmol/l 3-isobutyl-1-methylxanthine and 1 μmol/l indole indomethacin. Control cells were cultured in DMEM/F12 containing 10% fetal bovine serum. Cells were maintained at 37°C in a 5% CO_2_ incubator and the medium was changed every 3 days. Following a 3-week induction period, the cells were stained with alizarin red and cells exhibiting calcium nodule formation were observed under an inverted microscope (TMS; Nikon).

### Lentivirus packaging and quantitative fluorescence polymerase chain reaction (PCR)

Aliqots of 293T cells (GeneChem, Montreal, Canada) were cultured in high-glucose DMEM supplemented with 10% fetal bovine serum. Recombinant virus plasmid Ubi-CNGRC-3Flag-EGFP and packaging plasmids (Helper 1.0 and Helper 2.0) were prepared using an EndoFree Plasmid Maxi kit (Qiagen, Amsterdam, The Netherlands). Three plasmids were co-transfected into 293T cells using Lipofectamine 2000 (Life Technologies, Grand Island, NY, USA). After a 48 h transduction period, the supernatant containing lentiviral particles was harvested and concentrated by super-speed centrifugation at 4,000 × g for 10 min. Viral titers were measured by quantitative PCR, using GAPDH as an internal control. The Ct value was defined as the number of cycles when the fluorescent signal reached a specified threshold. The sequences of primers were as follows: EGFP, forward 5′-TGCTTCAGCCGCTACCC-3′ and reverse 5′-AGTTCACCTTGATGCCGTTC-3′; GAPDH, forward 5′-TGACTTCAACAGCGACACCCA-3′ and reverse 5′-CACCCTGTTGCTGTAGCCAAA-3′.

### Lentiviral infection of ADSCs

Concentrated lentiviral solutions of LV-EGFP and LV-CNGRC-3Flag-EGFP were added into two wells of cultured ADSCs, respectively, once the cells reached 40% confluence. Enhanced infection solution was then added to reach a total incubation volume of 2 ml. After 12 h of incubation, the cell culture medium was changed and 72 h later, images of the cells expressing EGFP were captured under an inverted fluorescence microscope (ECLIPSE Ti; Nikon). Images were processed using NIS-Elements imaging software (Nikon Instruments, Inc., Tokyo, Japan).

### Immunoblot analysis

Stable transfected ADSCs were selected with puromycin (Sigma-Aldrich, St. Louis, MO, USA) and CNGRC expression levels of cells at the third passage (12 days after transduction) were identified by western blot analysis. Total protein was extracted from ADSCs transfected with LV-EGFP and LV-CNGRC-3Flag-EGFP, respectively. Following denaturation, the protein samples were centrifuged at 12,000 × g for 2 min. The target proteins were hybridized with mouse anti-flag tag monoclonal antibody (1:1,000; Proteintech, Chicago, IL, USA), mouse anti-β-actin monoclonal antibody (1:5,000; Proteintech) and horseradish-peroxidase-conjugated goat anti-mouse IgG polyclonal antibody (1:10,000; CWBio, Beijing, China) IgG. Enhanced chemiluminescent substrates (Pierce Biotechnology, Inc., Rockford, IL, USA) were used to detect the signals of targeted proteins.

### Statistical analysis

All statistical analyses were performed using SPSS 16.0 software (SPSS, Inc., Chicago, IL, USA). Data are expressed as the mean ± standard error of the mean. The paired-sample t-test was used for comparisons among multiple groups and P<0.05 was considered to indicate a statistically significant difference.

## Results

### Successful isolation and culture of ADSCs

**Figure 1:**
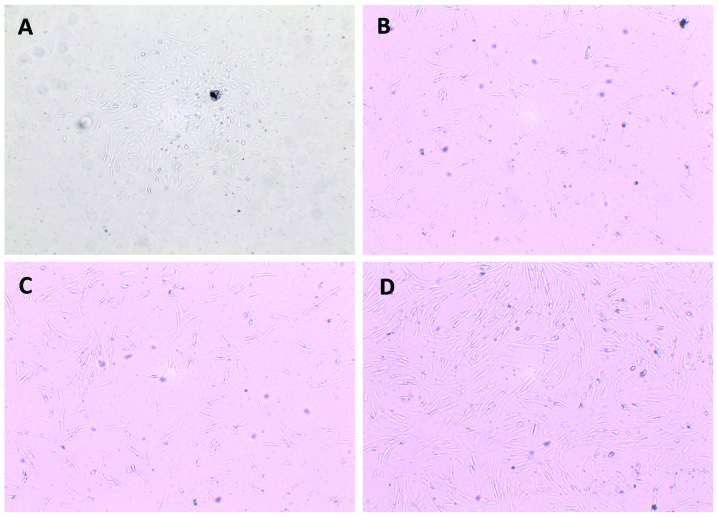
ADSCs at different passages observed under an inverted microscope. (A) Primary ADSCs cultured for 3 days (magnification, ×600). (B) ADSCs at passage 1 cultured for 11 days (magnification, ×600). (C) ADSCs at passage 3 cultured for 3 days (magnification, ×400). (D) ADSCs at passage 4 cultured for 3 days (magnification, ×400). ADSCs, adipose stem cells.

ADSCs were isolated from rat adipose tissue by use of enzymatic digestion. Twenty-four hours after seeding, several transparent adherent cells with round or oval morphology were visible under a microscope and at 4 days after seeding, additional ADSCs with fusiform and polygonal morphology were observed. At 10–11 days after seeding, the cell clones had significantly expanded, become connected to each other and reached 80–90% confluence. Cells with long spindle-shaped morphology were closely arranged in a swirling pattern with multilayer cells in the center. Following passage, the ADSCs demonstrated a higher growth rate. Without alterations in cell morphology, the doubling time of these cells was 70 h and the cells could be successfully sub-cultured (Fig. 1).

### ADSCs express typical surface markers in culture

**Figure 2:**
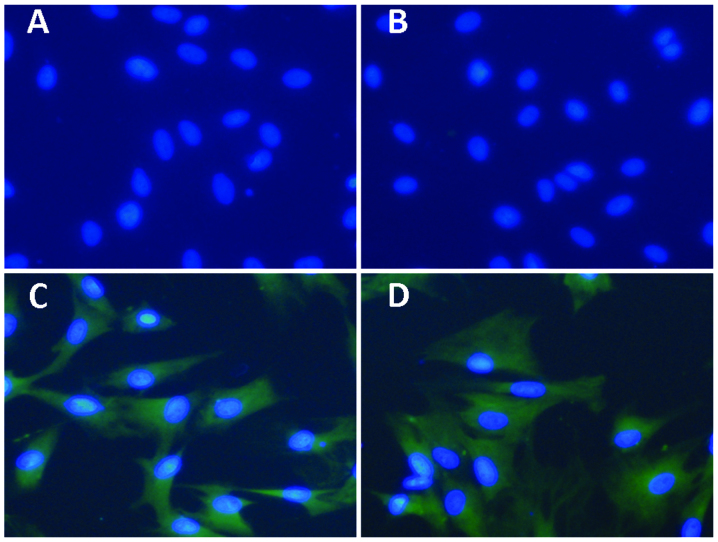
Surface markers expressed in ADSCs. Cultured ADSCs did not express (A) CD34 and (B) CD106, but did express (C) CD29 and (D) CD90. Green: surface markers. Blue: 4′,6-diamidino-2-phenylindole (magnification, ×2,000). ADSCs, adipose stem cells.

ADSCs were identified by cell surface markers (23). Previous studies reported that ADSCs expressed the cell surface markers CD90, CD29, CD49e, CD54, CD55, CD63, CD73 and CD105, but did not express CD11b, CD34, CD31, HLA-DR and CD117 (24,25). Zuk *et al* (26) revealed that ADSCs expressed CD49d but not CD106, while bone marrow mesenchymal stem cells expressed CD106 but not CD49d. In the present study, >80% of cultured cells were CD29 and CD90 double positive (Fig. 2C and D) and CD106 and CD34 double negative (Fig. 2A and B). These findings indicated that ADSCs were successfully isolated and cultured *in vitro*.

### ADSCs differentiate to form adipocytes in culture

**Figure 3:**
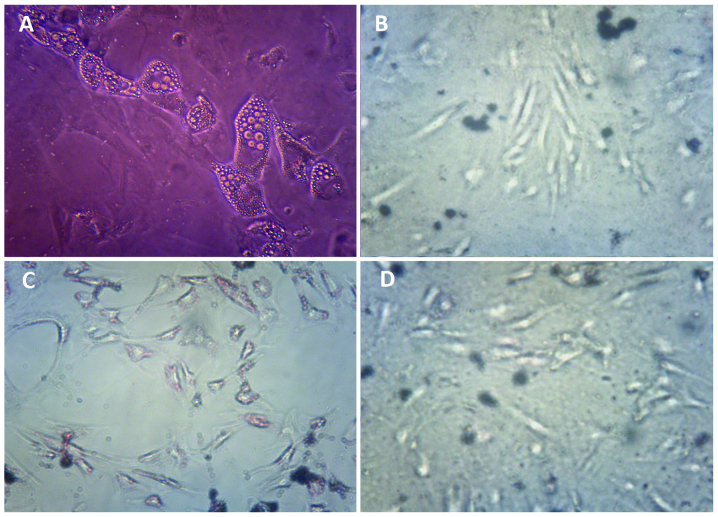
ADSCs following adipogenic induction. (A and B) ADSCs were cultured in induction medium (A, magnification, ×2,000) or normal medium (B, magnification, ×600) for 1 week. (C and D) Oil Red O staining-labeled fat droplets in ADSCs cultured in induction medium for 2 weeks (C, magnification, ×600) but not in cells cultured in normal medium (D, magnification, ×600). ADSCs, adipose stem cells.

Adipogenic inducers were added to cultures of ADSCs to determine their capacity for adipogenesis. Following induction for 24 h, the cells became smaller in size and the cytoplasm began to retract and demonstrated a small square morphology. The refractive index of these cells was also enhanced. At 3–4 days after induction, cell proliferation slowed significantly and small, round, shiny lipid droplets had formed in the cytoplasm. Observations made at longer time periods following induction revealed enlargement of lipid droplets in the cytoplasm and increased cell numbers (Fig. 3A and C). At 2 weeks after induction, ADSCs were stained with Oil Red O, which revealed the presence of various sized round red particles and fat droplets in the cytoplasm. By contrast, no significant difference was identified in cell size or formation of small lipid droplets in cells not treated with the adipogenic inducer (Fig. 3B and D).

### ADSCs differentiate to form osteocytes in culture

**Figure 4:**
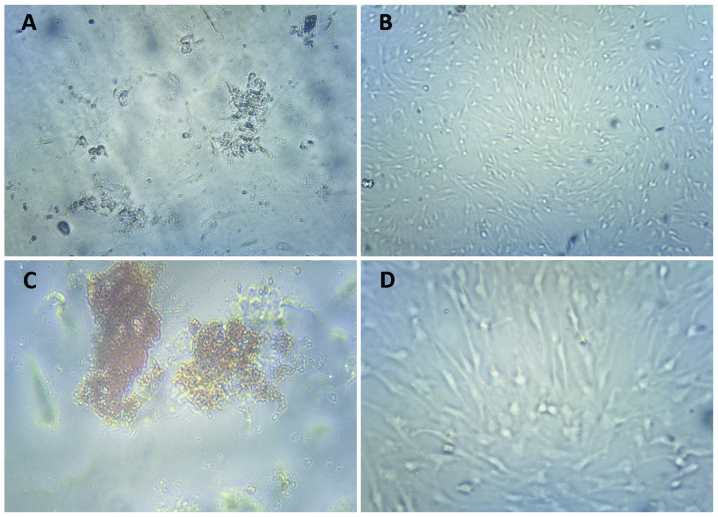
ADSCs following osteogenic induction. (A and B) ADSCs were cultured in induction medium (A, magnification, ×400) or normal medium (B, magnification, ×400) for 3 weeks. (C and D) Alizarin red stained calcium nodules in ADSCs cultured in induction medium (C, magnification, ×2,000) but not in cells cultured in normal medium (D, magnification, ×600). ADSCs, adipose stem cells.

Osteogenic inducers were added to cultures of ADSCs to examine their capacity for osteogenesis. After 3 days of induction, the morphology of ADSCs altered from a long spindle shape into a round or irregular shape. Additionally, cell body size increased, cellular projections disappeared and the cell proliferation rate decreased. One week after induction, the cells formed colonies, the nuclei became rounded and larger and additional small black particles appeared in the cytoplasm. Following longer periods of induction, the cells became distributed surrounding the colonies and grew in an overlapping manner. At 3 weeks after induction, the cells had secreted large quantities of extracellular matrix and formed growing granular calcium nodules of varying sizes (Fig. 4A). The mineralized nodules were distributed in colonies and had a reddish-brown color following alizarin red staining, confirming calcium deposition (Fig. 4C). By contrast, there was no calcium deposition in cells that were not subjected to induction (Fig. 4B and D).

### Expression of CNGRC in 293T cells and successful packaging of lentiviral vectors

**Figure 5:**
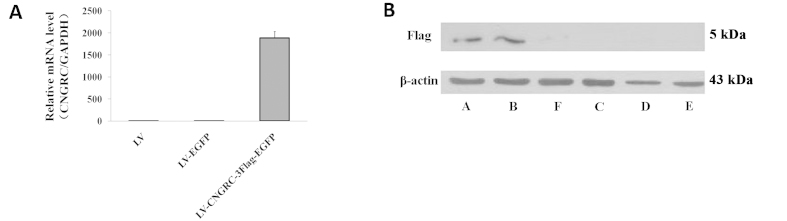
(A) CNGRC mRNA levels in ADSCs following transduction. Compared with non-transfected cells and cells transfected with LV-EGFP, cells transfected with LV-CNGRC-3Flag-EGFP contained significantly higher quantities of CNGRC mRNA. (B) Immunoblot showing expression of CNGRC peptide in (A, B and F) LV-CNGRC-3Flag-EGFP-transfected ADSCs, but not in (C and D) LV-EGFP-transfected ADSCs and (E) non-transfected cells. ADSCs, adipose stem cells.

Lentiviral vectors LV-EGFP and LV-CNGRC-3Flag-EGFP were co-transfected with Helper 1.0 and Helper 2.0 into 293T cells. CNGRC RNA levels in transfected and non-transfected cells were measured 72 h later by quantitative fluorescence PCR. CNGRC RNA levels in transfected cells were significantly higher (P<0.05) than levels in non-transfected and LV-EGFP-transfected cells (Table I and Fig. 5A), indicating successful expression of CNGRC in transfected cells. The titer of the lentivirus was calculated as 2.00E+8TU/ml, based on the difference in Ct values between non-transfected cells and transfected cells.

### Expression of exogenous DNA in ADSCs following lentivirus infection

**Figure 6:**
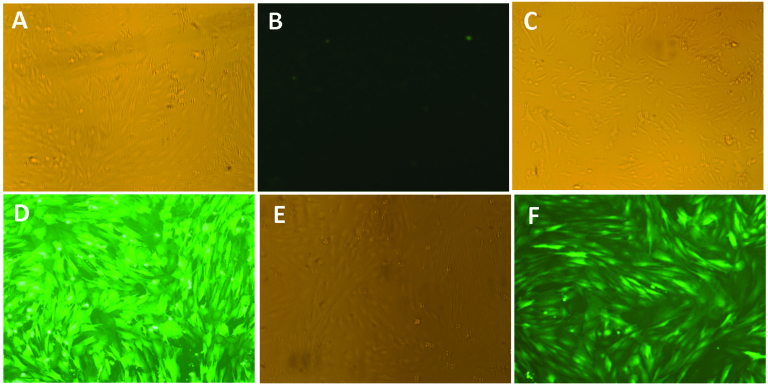
Expression of exogenous DNA in ADSCs following lentivirus infection. (A and B) Non-transfected ADSCs were observed under a (A) bright field and (B) fluorescence microscope. (C and D) LV-EGFP-transfected ADSCs were observed under a (C) bright field and (D) fluorescence microscope. (E and F) LV-CNGRC-3Flag-EGFP-transfected ADSCs were observed under a (E) bright field and (F) fluorescence microscope. Cells transfected with LV-EGFP and LV-CNGRC-3Flag-EGFP expressed high levels of EGFP, indicating successful transduction of lentivirus in cultured ADSCs. ADSCs, adipose stem cells.

To examine expression of exogenous DNA, transfected ADSCs were observed under a fluorescence microscope to detect the expression of EGFP after 72 h of transduction. LV-EGFP and LV-CNGRC-3Flag-EGFP demonstrated transduction efficiencies >80%. As shown in Fig. 6, EGFP was highly expressed in ADSCs transfected with the lentivirus. By contrast, no EGFP signal was detected in non-transfected cells. These results indicated that the exogenous gene could be expressed using lentiviral transduction.

### Expression of CNGRC peptide in ADSCs following lentiviral transduction

To confirm expression of the CNGRC peptide, total proteins were extracted from ADSCs transfected with LV-EGFP, LV-CNGRC-3Flag-EGFP and without transduction, respectively. CNGRC-3Flag-EGFP was detected at the expected size by western blot analysis using anti-flag antibody in LV-CNGRC-3Flag-EGFP transfected cells, but was not detected in either non-transfected or LV-EGFP-transfected cells (Fig. 5B). The results clearly demonstrated expression of CNGRC peptide in ADSCs at the third passage, 12 days after infection with the lentivirus.

## Discussion

ADSCs and bone marrow mesenchymal stem cells have similar phenotypes and differentiation potentials. However, it is easier to obtain large numbers of ADSCs than large numbers of mesenchymal stem cells (27). Bacigalupo *et al* (28) could obtain only 40 ml of human bone marrow under local anesthesia, which yielded ~1.2×10^5^ stem cells. To obtain larger quantities of bone marrow mesenchymal stem cells, surgery must be performed under whole-body anesthesia, which may cause other complications. By contrast, Aust *et al* (29) revealed that up to 4×10^6^ stem cells could be obtained from 200 ml of adipose tissue, which was ~30-fold greater than the number of mesenchymal stem cells obtained from bone marrow. In addition, previous studies have reported successful transduction of ADSCs using a lentivirus and identified that transduction efficiency was significantly greater when using ADSCs rather than bone marrow mesenchymal stem cells (30–32). In the present study, it was demonstrated that ADSCs could be easily isolated from rat adipose tissue and that transduction efficiency using lentiviral vectors was >80%. Additionally, DNA expressing CNGRC peptide was successfully transfected using lentiviral vectors.

Microvessels surrounding cancer stem cells (stem cell niche) are important in regulating cancer stem cell numbers and their functions (1,2). As mentioned previously, targeting of microvessels in stem cell niches has been suggested as an efficient method for treating tumors. NGR is a tri-peptide motif that can specifically bind to vascular endothelial cells of newly generated vessels in tumors (9). Based on this biological function, NGR-conjugates have been used in tumor diagnosis and treatment (12–17). However, the efficiency achieved when using this approach has been low due to the lack of a targeting vehicle. It was suggested that the efficient homing capacity of ADSCs could be exploited by using them as targeting vehicles to deliver NGR-conjugates specifically to cancer stem cell niches (3). To achieve this goal, it was first necessary to establish an ADSC cell line that stably expresses NGR-conjugates. In the present study, ADSC cell lines expressing the CNGRC peptide were established using lentiviral transduction. The mechanism by which these CNGRC-expressing ADSC cell lines migrate to cancer stem cell niches and eliminate newly generated microvessels remains to be elucidated. Future studies are to be conducted to assess the homing capacity of these ADSC cell lines and the *in vivo* vascular tropism effects of the CNGRC peptide.

## Figures and Tables

**Table I:** Relative quantities of CNGRC mRNA in non-transfected, LV-EGFP-transfected and LV-CNGRC-3Flag-EGFP-transfected adipose stem cells.

Relative mRNA level	LV^a^	LV-EGFP^b^	LV-CNGRC-3Flag-EGFP^a^^b^
(Average ± STDEV)	1.054±0.413	0.967±0.538	1883.897±145.304

P<0.05, LV-CNGRC-EGFP vs. LV; P<0.05, LV-CNGRC-EGFP vs. LV-EGFP; STDEV, standard deviation; LV, lentivirus; EGFP, enhanced green fluorescent protein.

a LV-CNGRC-3Flag -EGFP vs. LV; P < 0.05.

b LV-CNGRC-3Flag -EGFP vs. LV-EGFP; P<0.05.
